# Large Language Models for World Health Organization–Uppsala Monitoring Centre Drug–Adverse Event Causality Assessment Using Food and Drug Administration Adverse Event Reporting System Cases: Comparative Performance Study

**DOI:** 10.2196/93237

**Published:** 2026-07-08

**Authors:** Young Mi Ha, Minjung Kim, YoungIn Bang, Daejin Choi, Jae Hyun Kim, Sandy Jeong Rhie, Yoshihiro Noguchi, Myeong Gyu Kim

**Affiliations:** 1 Graduate School of Pharmaceutical Sciences Ewha Womans University Seoul Republic of Korea; 2 College of Pharmacy Ewha Womans University Seoul Republic of Korea; 3 College of Artificial Intelligence Ewha Womans University Seoul Republic of Korea; 4 Human-Centered Artificial Intelligence Research Institute Ewha Womans University Seoul Republic of Korea; 5 School of Pharmacy and Institute of New Drug Development Jeonbuk National University Jeonju Republic of Korea; 6 Laboratory of Clinical Pharmacy Gifu Pharmaceutical University Gifu Japan

**Keywords:** large language models, World Health Organization–Uppsala Monitoring Centre causality assessment, WHO-UMC causality assessment, prompt engineering, generative pretrained transformer, GPT, Gemini

## Abstract

**Background:**

Causality assessment is central to pharmacovigilance but remains resource-intensive and subjective. The applicability of large language models (LLMs) to formal World Health Organization–Uppsala Monitoring Centre (WHO-UMC) drug–adverse event causality assessment has not been well established.

**Objective:**

This study aims to evaluate the performance of LLMs in WHO-UMC causality assessment.

**Methods:**

A curated set of 55 cases derived from the US Food and Drug Administration Adverse Event Reporting System, comprising 337 drug-level assessments, was constructed. Cases involving 2 to 11 suspected drugs were stratified by drug count, and 5 cases were sampled from each stratum. To ensure representation of rare but clinically important categories, 5 additional cases containing at least 1 “Certain” drug–adverse event pair were included. Case data were reorganized into a standardized semistructured format that preserved key elements required for WHO-UMC causality assessment. Domain experts conducted a pilot evaluation to align interpretation criteria prior to independently assessing the final dataset, yielding an interexpert agreement (Fleiss κ) of 0.762 across 337 drug-level assessments. Multiple prompting strategies, including standard prompting, chain-of-thought (CoT), CoT with self-consistency, few-shot, reasoning and acting, and tree-of-thought prompting, were applied across multiple LLMs, including GPT-5.4 and its mini variant and Gemini 2.5 Flash and Pro, via their respective application programming interfaces. Agreement with expert assessments was quantified using Cohen κ, weighted κ, and accuracy metrics. Internal consistency across repeated inferences was evaluated using Fleiss κ.

**Results:**

Performance varied across models and prompting strategies. Cohen κ ranged from 0.368 to 0.641, weighted κ ranged from 0.641 to 0.821, accuracy ranged from 0.583 to 0.804, and balanced accuracy ranged from 0.513 to 0.735. Fleiss κ ranged from 0.730 to 0.915, corresponding to substantial to almost perfect agreement. The highest Cohen κ was observed for Gemini 2.5 Flash with CoT prompting (0.641). Gemini 2.5 Flash with CoT–self-consistency prompting showed a Cohen κ of 0.640 and achieved the highest observed point estimates for weighted κ (0.821), accuracy (0.804), and Fleiss κ (0.915), although the gains over other prompting strategies were modest. Category-level performance for this model showed higher performance for “Certain” (*F*_1_-score=0.793), “Probable/Likely” (*F*_1_-score=0.794), and “Unlikely” (*F*_1_-score=0.898), whereas performance for “Possible” remained substantially lower (*F*_1_-score=0.293), reflecting the difficulty of intermediate causality assessment.

**Conclusions:**

LLMs demonstrated moderate to substantial agreement in WHO-UMC causality assessment, indicating meaningful but still limited performance relative to expert judgment. Although LLMs are not suitable for independent decision-making, they may serve as supportive tools in pharmacovigilance workflows, particularly for preliminary case triage. Further studies using larger and more diverse datasets and evaluating performance on raw narrative reports are warranted.

## Introduction

Postmarketing pharmacovigilance plays a critical role in identifying and characterizing adverse drug reactions that may not be fully captured during preapproval clinical trials [1,2]. Among various pharmacovigilance activities, causality assessment between drugs and reported adverse events (AEs) is essential for signal detection, regulatory decision-making, and patient safety [3,4]. However, causality assessment remains inherently complex due to factors such as polypharmacy, comorbidities, incomplete clinical information, and variability in expert judgment [5,6]. Spontaneous reporting systems, including the US Food and Drug Administration Adverse Event Reporting System (FAERS), provide large-scale real-world data for postmarketing safety surveillance [1]. Despite their value, FAERS reports are heterogeneous in quality and often lack detailed clinical context, further complicating structured causality assessment at scale.

The World Health Organization–Uppsala Monitoring Centre (WHO-UMC) causality assessment system is one of the most widely used frameworks for evaluating drug-AE relationships in spontaneous reports [3,7]. Although the WHO-UMC system provides standardized criteria, its application relies heavily on expert interpretation, which introduces subjectivity and interrater variability [3,4]. Moreover, expert-driven causality assessment is time-consuming and resource-intensive, making it difficult to apply consistently across large datasets such as FAERS [8]. These limitations highlight the need for scalable, reproducible approaches that can support or augment expert judgment without compromising methodological rigor.

Recent advances in large language models (LLMs) have demonstrated their potential to perform complex reasoning tasks across biomedical domains, including clinical text interpretation, medical decision support, and regulatory documentation [9,10]. LLMs are particularly well-suited for analyzing unstructured or semistructured text such as spontaneous AE reports. Several studies have explored the use of LLMs in AE detection tasks [11-13]; however, systematic evaluations of LLMs for formal drug-AE causality assessment remain limited. In particular, there is a lack of evidence regarding whether LLMs can reliably reproduce expert-level WHO-UMC causality classifications and how their performance varies depending on prompt design, model configuration, and inference stability.

Given these considerations, a rigorous evaluation framework is required to assess LLM performance in drug-AE causality assessment. This framework should incorporate carefully curated FAERS cases suitable for WHO-UMC assessment, expert consensus as a reference standard, systematic comparisons of prompt engineering strategies, cross-model evaluations across different LLM architectures and configurations, and quantitative agreement metrics (including κ statistics), along with analyses of response consistency.

The objective of this study was to systematically evaluate the performance of LLMs in drug-AE causality assessment using the WHO-UMC framework, based on structured FAERS cases. Specifically, this study aimed to examine different prompt engineering strategies; assess multiple LLM configurations, including GPT-5.4, GPT-5.4 mini, Gemini 2.5 Pro, and Gemini 2.5 Flash; and examine the internal consistency of LLM outputs across repeated assessments.

## Methods

**Figure 1:**
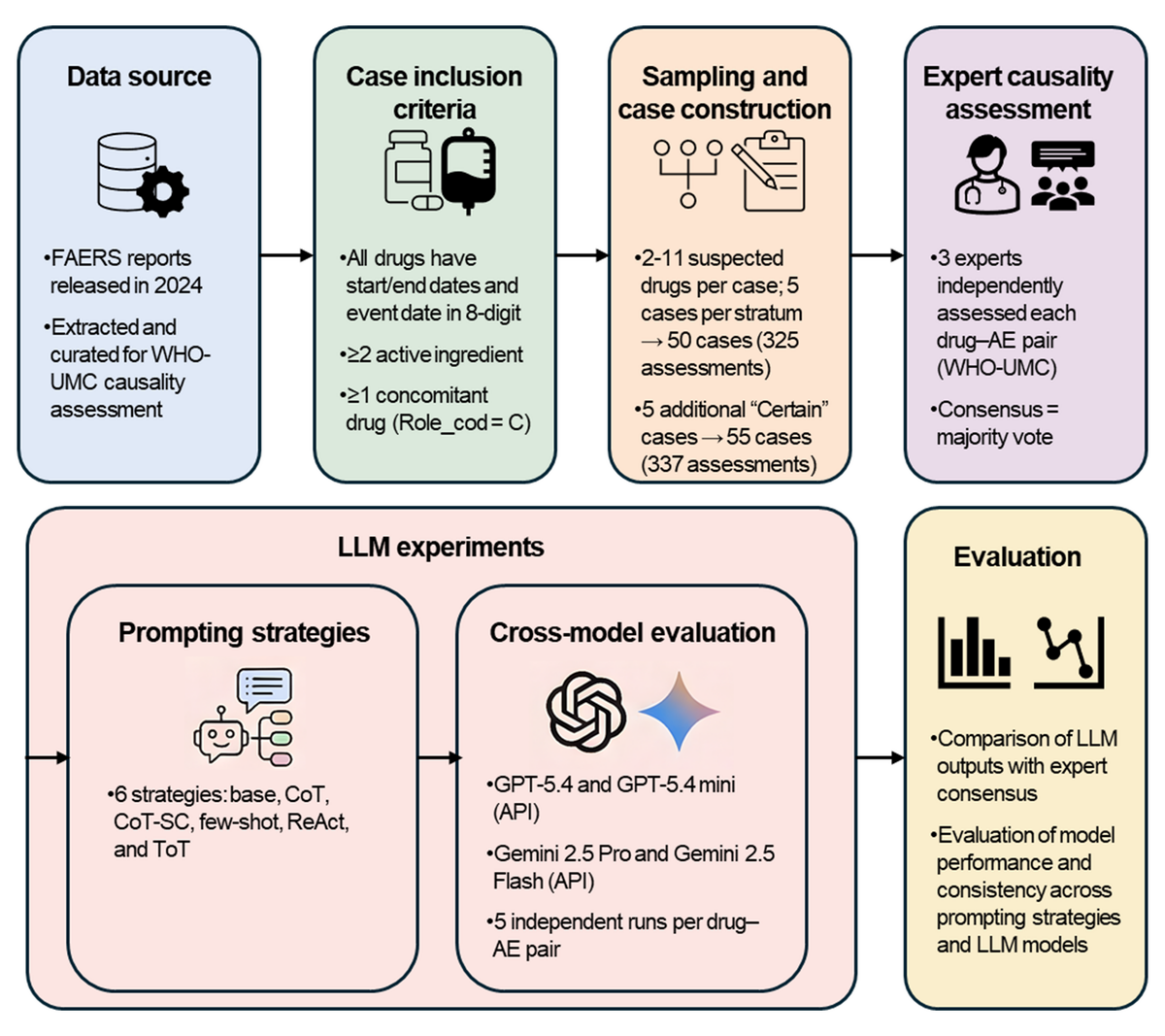
Study workflow for evaluating large language model (LLM)–based World Health Organization–Uppsala Monitoring Centre (WHO-UMC) causality assessment. Food and Drug Administration Adverse Event Reporting System (FAERS) reports released in 2024 were curated and filtered to construct 55 drug–adverse event (AE) cases comprising 337 drug-level assessments. Three experts independently assessed each drug-AE pair using the WHO-UMC framework, and the reference standard was determined by majority vote. LLM experiments included 6 prompting strategies (base, chain-of-thought [CoT], CoT–self-consistency [SC], few-shot, reasoning and acting [ReAct], and tree-of-thought [ToT]) and 4 model configurations (GPT-5.4, GPT-5.4 mini, Gemini 2.5 Pro, and Gemini 2.5 Flash). Each model configuration was evaluated using 5 independent runs per drug-AE pair. Model outputs were compared against expert consensus to assess performance and consistency. API: application programming interface.

Figure 1 presents an overview of the study workflow.

### Data Source and Case Construction

This study was conducted using data derived from the FAERS, using reports released in 2024 as the source dataset. Relevant information required for drug-AE causality assessment was extracted and curated into structured analytic cases.

Cases were selected based on predefined inclusion criteria to ensure suitability for causality assessment. Eligible cases were required to (1) contain clearly documented start and end dates in an 8-digit format (YYYYMMDD) for all reported drugs, (2) include drugs with at least 2 active ingredient names, and (3) report at least 1 drug classified as concomitant (Role_cod=C) among the reported medications.

Sample size requirements were determined a priori based on agreement analysis using the κ statistic. The null hypothesis κ was set at 0.30, representing the midpoint of fair agreement, and the alternative hypothesis κ was set at 0.50, representing the midpoint of moderate agreement. Assuming 6 WHO-UMC causality categories (“Certain,” “Probable/Likely,” “Possible,” “Unlikely,” “Conditional/Unclassified,” and “Unassessable/Unclassifiable”) with imbalanced category distributions, a 2-sided α level of 0.05, and 90% statistical power, a minimum of 292 drug-level assessments was required [14].

In the source FAERS database, cases involving 2 to 11 suspected drugs accounted for approximately 90% of all reports; therefore, this range was selected for analysis. To control for complexity related to the number of concomitant drugs, cases were stratified by drug count, and an equal number of cases was sampled from each stratum. Sampling 5 cases per stratum yielded a total of 50 cases, corresponding to 325 drug-level assessments, which exceeded the minimum required sample size.

Notably, no drug-AE pairs classified as “Certain” were identified in the initial sample, and no cases were assigned to the “Conditional/Unclassified” category. Given the clinical importance and distinct evidentiary requirements of the “Certain” classification, additional cases containing at least 1 drug-AE pair with evidence of a positive rechallenge, which is required for a “Certain” classification, were retrieved (6228 out of 1,484,350 cases in 2024; 0.4% of total cases). Among these, 30 (0.5%) cases met the predefined inclusion criteria for this study. These candidate pairs were subsequently reviewed by experts to confirm the “Certain” classification. From this pool, 5 (16.7%) cases containing at least 1 confirmed “Certain” drug-AE pair were randomly selected to ensure representation of this rare but clinically important category while maintaining balance with the original sampling framework. Consequently, the final dataset comprised 55 cases and 337 drug-level assessments.

All cases were structured to enable causality assessment in accordance with the WHO-UMC causality assessment criteria. Data were obtained from the “DEMOGRAPHIC,” “DRUG,” “REACTION,” “THERAPY,” and “INDICATIONS” files of the FAERS database and merged using the unique report identifier (PRIMARYID). From these files, the researchers extracted patient demographic variables (age, age unit, and sex), drug-related information (active ingredient, dose, route, indication, and therapy start and end dates), AEs coded as Medical Dictionary for Regulatory Activities preferred terms, event date, and dechallenge or rechallenge information.

The extracted data were subsequently reorganized into a standardized textual format to enable consistent input to the LLM. This preprocessing step was limited to restructuring and consolidating dispersed fields into a unified representation (eg, patient summary, medication list, and event timeline), without reinterpretation, summarization, or exclusion of clinically relevant information from the original reports. Drug identifiers (eg, “Drug1” and “Drug2”) were introduced solely to distinguish multiple entries within a case, while retaining the corresponding active ingredient information. All cases reformatted into the input structure used for LLM evaluation are provided in Multimedia Appendix 1.

Although the researcher responsible for case selection also participated in the expert-based causality assessment, all cases were selected using predefined criteria and random sampling procedures to minimize potential selection bias, and no information regarding case composition was shared during the assessment process.

### Expert-Based Causality Assessment

Causality assessment for each drug-AE pair was independently performed by 3 domain experts with experience in pharmacovigilance and adverse drug reaction evaluation, with each expert blinded to the assessments of the others. Assessments were conducted in accordance with the WHO-UMC causality assessment system.

Prior to the main analysis, a pilot evaluation was conducted using a separate set of cases not included in the main analysis to assess interexpert agreement and ensure adequate consistency among the expert raters. The pilot was designed to achieve a predefined level of agreement, defined as a Fleiss κ of at least 0.50, corresponding to the midpoint of moderate agreement, and the absence of drug-AE pairs for which all 3 experts assigned completely discordant categories. An initial pilot was conducted using 5 randomly selected cases (46 drug-level assessments), but the predefined criteria were not met. Following this, discrepancies were reviewed, and consensus discussions were undertaken to harmonize the interpretation of the WHO-UMC criteria. A second pilot using an additional 5 cases (42 drug-level assessments) achieved the target agreement threshold, with a Fleiss κ of 0.641, and satisfied the predefined criteria. The main analysis was subsequently conducted on the final dataset, yielding an interexpert agreement (Fleiss κ) of 0.762 across 337 drug-level assessments.

For the primary outcome analysis, the expert reference standard was defined using a majority vote approach across the 3 experts for each drug-AE pair. In instances where a majority decision could not be reached, the case was independently evaluated by a fourth expert, whose assessment was used to determine the final causality classification.

### Prompt Engineering Strategies

The prompts used for causality assessment were initially drafted using ChatGPT (version 5.0 [web version]; OpenAI) and subsequently refined by the researchers to ensure alignment with the WHO-UMC causality assessment criteria and the objectives of this study.

The final prompt consisted of 3 main components: (1) general instructions defining the role of the model as a pharmacovigilance expert and specifying independent evaluation of each drug-AE pair; (2) critical interpretation guidance to standardize decision-making across key domains such as temporal relationship, pharmacological plausibility, alternative explanations, and dechallenge or rechallenge information; and (3) a structured output format requiring categorical classification according to WHO-UMC criteria along with a concise reasoning summary.

To evaluate different prompt engineering strategies, multiple prompting approaches were implemented, including standard prompting (base), chain-of-thought (CoT), CoT with self-consistency (SC), few-shot, reasoning and acting, and tree-of-thought prompting. The full prompt templates used in this study are provided in Multimedia Appendix 2.

### Performance Comparison of LLMs Across Prompting Strategies

All model evaluations were conducted via application programming interfaces. The following models were used: GPT-5.4 (gpt-5.4-2026-03-05) and GPT-5.4 mini (gpt-5.4-mini-2026-03-17) from OpenAI, as well as Gemini 2.5 Pro (gemini-2.5-pro) and Gemini 2.5 Flash (gemini-2.5-flash) from Google DeepMind, for which the latest update was in June 2025 with a knowledge cutoff of January 2025.

For each drug-AE pair and prompting strategy, model inference was repeated 5 independent times to account for potential variability in model outputs. The choice of 5 repetitions was informed by prior prompt-repetition studies and reflects a practical trade-off between capturing probabilistic output variability and limiting computational overhead [15]. To reduce stochastic variability, the temperature parameter was fixed at 0 for all runs, while all other generation parameters (eg, top_p) were left at the default settings of each provider and were not standardized across providers.

All prompts were identical across models except for the specified prompting strategies, ensuring a fair comparison. In addition, inference time for each application programming interface call was recorded by measuring the elapsed time between request initiation and response completion.

The final performance metrics, including Cohen κ, weighted κ, accuracy, balanced accuracy, and Fleiss κ, were calculated based on the results of the 5 independent runs, with mean values and 95% bootstrap CIs (1000 resamples) estimated across runs.

### Statistical Analysis

Performance was evaluated using Cohen κ, weighted κ, accuracy, and balanced accuracy [16,17]. Weighted κ coefficients were calculated to account for the ordinal nature of the WHO-UMC causality categories, including “Certain,” “Probable/Likely,” “Possible,” and “Unlikely.” Nonordinal categories, including “Conditional/Unclassified” and “Unassessable/Unclassifiable,” were excluded from the weighted κ calculations. Cohen κ, weighted κ, accuracy, and balanced accuracy were calculated across 5 independent runs, and the results are presented as mean values with corresponding 95% bootstrap CIs. For the estimation of balanced accuracy, stratified bootstrap sampling was applied to preserve class proportions within each resampled dataset. Internal consistency of LLM outputs across repeated runs was evaluated by calculating Fleiss κ for each model-prompt combination using the 5 repeated assessments [18].

Systematic differences between LLM-based and expert-based causality classifications were examined using confusion matrices constructed for each model. These matrices were further analyzed to identify patterns of misclassification, including tendencies toward overestimation or underestimation of causality within specific WHO-UMC categories. In addition, class-wise performance metrics, including recall, precision, specificity, and *F*_1_-score, were calculated based on the confusion matrix of the best-performing model configuration to further characterize category-level performance.

### Ethical Considerations

This study used publicly available data from the FAERS. The dataset is fully deidentified and does not contain personally identifiable information. This study received an exemption determination from the institutional review board of Ewha Womans University (ewha-202502-0005-01). No informed consent was required as the study involved secondary analysis of anonymized data. No participants were recruited, and no compensation was provided. The study does not include any identifiable images or personal data.

## Results

### Characteristics of the Study Cases and Drug-AE Pairs

Table 1 summarizes the characteristics of the evaluated cases, drugs, AEs, and expert-assigned WHO-UMC causality classifications. Among the 55 cases, sex was relatively evenly distributed between male (n=23, 41.8%) and female (n=26, 47.3%) participants, with 10.9% (n=6) classified as unknown. Most patients were aged 18 to 64 years (n=26, 47.3%) or ≥65 years (n=21, 38.2%), with smaller proportions aged <18 years (n=2, 3.6%) or with unknown age (n=6, 10.9%).

At the drug level (n=337), antineoplastic and immunomodulating agents (Anatomical Therapeutic Chemical Classification System group L) were the most frequently involved drugs (n=135, 40.1%), followed by agents affecting the nervous system (n=38, 11.3%), blood and blood-forming organs (n=36, 10.7%), and alimentary tract and metabolism agents (n=35, 10.4%). A total of 203 AEs were analyzed, most commonly involving blood and lymphatic system disorders (n=25, 12.3%) and gastrointestinal disorders (n=19, 9.3%).

According to expert consensus using the WHO-UMC system, 1.5% (n=5) of drug-AE pairs were classified as “Certain,” while the majority were categorized as “Unlikely” (n=205, 60.8%), followed by “Probable/Likely” (n=87, 25.8%) and “Possible” (n=39, 11.6%). Only 1 (0.3%) case was deemed “Unassessable/Unclassifiable,” and no cases were classified as “Conditional/Unclassified.”

**Table 1:** Characteristics of study cases and drug–adverse event pairs based on a sampled subset of 2024 Food and Drug Administration Adverse Event Reporting System reports.

Characteristics				Participants, n (%)
**Case-level characteristics (n=55)**				
	**Sex**			
		Male	23 (41.8)	
		Female	26 (47.3)	
		Unknown	6 (10.9)	
	**Age groups (years)**			
		<18	2 (3.6)	
		18-64	26 (47.3)	
		≥65	21 (38.2)	
		Unknown	6 (10.9)	
**Drug-level characteristics (n=337)**				
	**Anatomical Therapeutic Chemical Classification System anatomical group (level 1)**			
		L—antineoplastic and immunomodulating agents	135 (40.1)	
		N—nervous system	38 (11.3)	
		B—blood and blood-forming organs	36 (10.7)	
		A—alimentary tract and metabolism	35 (10.4)	
		J—anti-infectives for systemic use	26 (7.7)	
		Others	67 (19.9)	
	**World Health Organization–Uppsala Monitoring Centre causality classification (expert consensus)**			
		Certain	5 (1.5)	
		Probable or likely	87 (25.8)	
		Possible	39 (11.6)	
		Unlikely	205 (60.8)	
		Conditional or unclassified	0 (0)	
		Unassessable or unclassifiable	1 (0.3)	
**Adverse event characteristics (n=203): Medical Dictionary for Regulatory Activities system organ class**				
	Blood and lymphatic system disorders		25 (12.3)	
	Gastrointestinal disorders		19 (9.3)	
	Injury, poisoning, and procedural complications		16 (7.9)	
	General disorders and administration site conditions		16 (7.9)	
	Infections and infestations		16 (7.9)	
	Respiratory, thoracic, and mediastinal disorders		14 (6.9)	
	Others		97 (47.8)	

### Performance Comparison of LLMs Across Prompting Strategies

Performance varied across models and prompting strategies (Multimedia Appendix 3). Across all evaluated configurations, Cohen κ ranged from 0.368 to 0.641, weighted κ ranged from 0.641 to 0.821, accuracy ranged from 0.583 to 0.804, and balanced accuracy ranged from 0.513 to 0.735, indicating variability in performance depending on both model and prompt design. Fleiss κ ranged from 0.730 to 0.915, corresponding to levels typically interpreted as substantial to almost perfect agreement, and suggesting relatively high internal consistency across repeated assessments. The longest observed inference time was 105.4 seconds (Gemini 2.5 Pro), with all responses completed within 2 minutes.

Among the evaluated configurations, the highest Cohen κ was observed for Gemini 2.5 Flash with CoT prompting (Cohen κ=0.641). Gemini 2.5 Flash with CoT-SC prompting showed a nearly identical Cohen κ (0.640) and achieved the highest observed point estimates for several other metrics, including weighted κ (0.821), accuracy (0.804), and Fleiss κ (0.915; Table 2). However, the improvement over the base prompt was modest, and the 95% CIs for key metrics overlapped substantially. A similar pattern was observed for Gemini 2.5 Pro, for which CoT-SC prompting showed the highest observed Cohen κ (0.514), although the differences among prompting strategies were modest and the 95% CIs substantially overlapped.

**Table 2:** Performance comparison of large language models using their best-performing prompting strategies.

Models^a^	Prompting strategies	Cohen κ	Weighted κ	Accuracy	Balanced accuracy	Fleiss κ
Gemini 2.5 Pro	CoT^b^-SC^c^	0.514	0.700	0.704	*0.735* ^d^	0.758
Gemini 2.5 Flash	CoT-SC	*0.640*	*0.821*	*0.804*	0.723	*0.915*
GPT-5.4	Few-shot	0.495	0.728	0.686	0.716	0.884
GPT-5.4 mini	CoT	0.435	0.651	0.661	0.538	0.820

^a^For each model, results correspond to the best-performing prompt among multiple prompt configurations.

^b^CoT: chain-of-thought.

^c^SC: self-consistency.

^d^Italicized values indicate the highest score for each metric across models.

### Agreement Patterns Across WHO-UMC Categories

**Figure 2:**
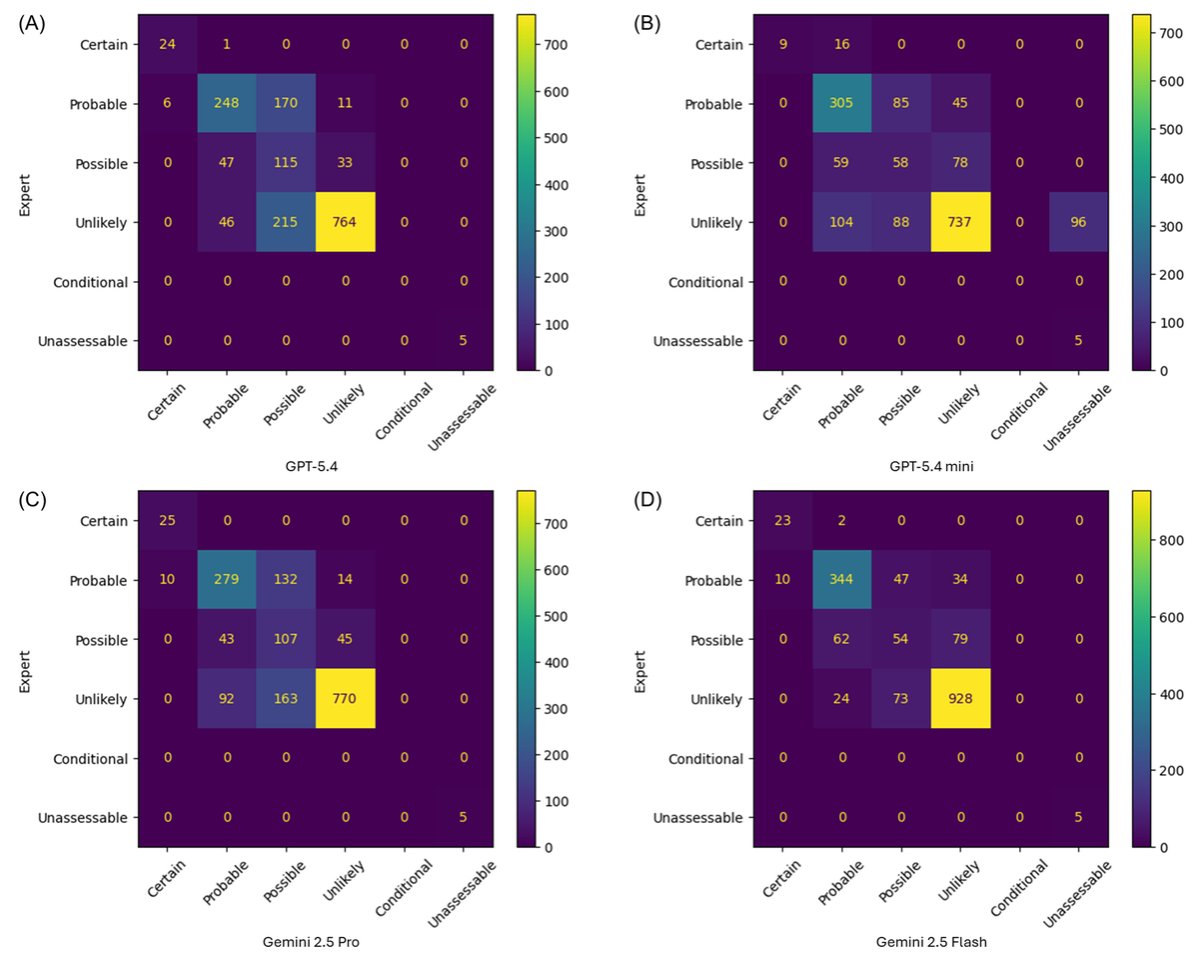
Confusion matrices comparing large language model (LLM)–based and expert-based World Health Organization–Uppsala Monitoring Centre (WHO-UMC) causality assessments across model configurations: (A) GPT-5.4, (B) GPT-5.4 mini, (C) Gemini 2.5 Pro, and (D) Gemini 2.5 Flash. Rows represent expert assessments, and columns represent LLM predictions. Each cell indicates the frequency of classifications at the drug–adverse event pair level, aggregated across 5 independent runs (ie, counts are summed across runs). Color intensity reflects the number of instances. WHO-UMC categories include “Certain,” “Probable/Likely,” “Possible,” “Unlikely,” “Conditional/Unclassified,” and “Unassessable/Unclassifiable.”.

Confusion matrices illustrating the agreement between model outputs and expert assessments across WHO-UMC categories are presented in Figure 2. Across models, correct classifications were generally concentrated along the diagonal, indicating that the models were broadly able to distinguish between causality categories. Misclassification patterns were primarily observed between adjacent categories, most notably with “Possible” cases being classified as either “Probable/Likely” or “Unlikely.”

For the best-performing configuration, Gemini 2.5 Flash (Figure 2), correct classifications were most prominent in the “Unlikely” category, with 928 (90.5%) assessments correctly identified. High agreement was also observed for “Probable/Likely” (n=344, 79.1% assessments) and “Certain” (n=23, 92% assessments). In contrast, the “Possible” category showed comparatively lower agreement, with substantial misclassification into both “Probable/Likely” (n=62, 31.8% assessments) and “Unlikely” (n=79, 40.5% assessments), indicating difficulty in resolving borderline causality assessments. Misclassification of “Probable/Likely” cases into adjacent categories was also observed but to a lesser extent.

Class-wise performance metrics for Gemini 2.5 Flash further supported these patterns (Table 3). The “Unlikely” category demonstrated high recall (0.905) and precision (0.891), resulting in the highest overall *F*_1_-score (0.898), indicating robust performance in identifying low-probability causality. The “Certain” category showed high recall (0.920) but relatively lower precision (0.697), suggesting occasional overclassification into this category. The “Probable/Likely” category demonstrated balanced performance (recall=0.791 and precision=0.796), reflecting moderate agreement with expert assessments.

In contrast, the “Possible” category exhibited substantially lower recall (0.277) and precision (0.310), consistent with the observed confusion with adjacent categories in the confusion matrix. The “Unassessable/Unclassifiable” category, although limited in number (n=5, 0.3%), was consistently identified with perfect agreement. The “Conditional/Unclassified” category was not represented in the dataset and was therefore not evaluated.

**Table 3:** Class-wise performance metrics of Gemini 2.5 Flash for World Health Organization–Uppsala Monitoring Centre causality assessment.

Class	Recall	Precision	Specificity	*F*_1_-score
Certain	0.920	0.697	0.994	0.793
Probable or likely	0.791	0.796	0.930	0.794
Possible	0.277	0.310	0.919	0.293
Unlikely	0.905	0.891	0.829	0.898
Conditional or unclassified^a^	—^b^	—	1.000	—
Unassessable or unclassifiable^c^	1.000	1.000	1.000	1.000

^a^“Conditional or unclassified” category contained no instances in the evaluation dataset; therefore, recall, precision, and *F*_1_-score were not defined.

^b^Not available.

^c^Metrics for the “Unassessable or unclassifiable” category were derived from 1 true case assessment repeated across inference runs; therefore, the perfect scores for this category should be interpreted with caution.

## Discussion

### Principal Findings

This study systematically evaluated the performance of LLMs in drug-AE causality assessment using the WHO-UMC framework, with a focus on the effects of prompt engineering strategies and model configurations. Overall, performance varied across models and prompting approaches, with the highest observed point estimates obtained for Gemini 2.5 Flash under CoT-SC prompting. However, the differences between prompting strategies were modest, and the CIs overlapped substantially, suggesting that these findings should be interpreted as exploratory rather than as evidence of the definitive superiority of this configuration.

A consistent pattern across analyses was that model performance differed by causality category. Categories with clearer decision boundaries, such as “Unlikely” and “Certain,” showed relatively high agreement with expert assessments. In contrast, intermediate categories, particularly “Possible,” demonstrated substantially lower recall and precision, with confusion matrix analysis indicating frequent misclassification into adjacent categories, most notably “Probable/Likely” and “Unlikely.” This pattern is consistent with the intrinsic characteristics of the “Possible” category within the WHO-UMC framework, which by definition accommodates incomplete information and competing explanations and is known to exhibit considerable interexpert variability even among human assessors.

The variability observed across repeated assessments was quantified using Fleiss κ, which fell within ranges typically interpreted as substantial to almost perfect agreement. This finding suggests that, under controlled inference settings, LLM outputs were relatively consistent across runs. Nevertheless, some variability remained, indicating that deterministic decoding (temperature=0) does not fully eliminate differences in model outputs. Notably, the run-to-run agreement of repeated LLM inferences, as measured by Fleiss κ, ranged from 0.730 to 0.915; for reference, the interexpert agreement in this study was 0.762. From a pharmacovigilance perspective, this finding suggests that LLM-based causality assessments exhibit a degree of variability similar to that seen in expert evaluations.

In this study, 5 independent runs were performed to assess the methodological robustness and variability of model performance by averaging the resulting metrics. However, in real-world pharmacovigilance workflows, the system could be operationalized to provide a definitive causality label through a single inference run for efficiency. For applications requiring enhanced reliability, a multirun strategy could also be implemented together with a predefined tie-breaking protocol. For example, cases with inconclusive outputs, such as a 2:2:1 split, could be resolved through additional iterative runs or flagged for manual expert adjudication. This approach would allow the system to balance computational efficiency, methodological robustness, and practical decisiveness.

From a regulatory science and pharmacovigilance perspective, these findings suggest that LLMs are better positioned as tools to support, rather than replace, expert judgment. Although agreement levels reached moderate to substantial ranges depending on the metric, they remain below typical expert-level concordance, indicating that misclassification persists, particularly in intermediate categories such as “Possible.” In this context, LLMs may be effectively applied to the initial triage of large-scale FAERS cases, prioritizing reports classified as “Certain,” “Probable/Likely,” or “Unlikely.” However, cases classified by LLMs as “Possible” should be subject to careful expert review to ensure appropriate interpretation and regulatory decision-making.

Within this framework, suspect drugs designated in FAERS are subsequently used as the basis for signal detection through disproportionality analysis, and this study’s focus on drug-level causality assessment provides a basis for examining how LLM-guided identification of suspect drugs may influence downstream signal detection. Therefore, future research could compare disproportionality analysis results obtained using drugs identified as suspect through LLM-based causality assessment with those derived from conventionally designated suspect drugs in FAERS, helping to clarify whether and how LLM-guided causality assessment can complement existing pharmacovigilance workflows.

### Comparison With Prior Work

Several recent studies have explored the application of LLMs to causality assessment; however, these efforts differ substantially in scope and methodological rigor from this work. Pandya et al [19] reported a case study applying ChatGPT to toxic epidermal necrolysis attributed to rifampicin and isoniazid. In that report, causality assessment using the WHO-UMC scale by human experts classified both drugs as “Probable,” whereas ChatGPT concluded that there was a “strong likelihood of a causal relationship.” However, ChatGPT did not provide a clear or structured causal analysis, and substantial numerical inaccuracies were observed in severity scoring, with marked overestimation of both the Score for Toxic Epidermal Necrolysis and ABCD-10 (age, bicarbonate, cancer, dialysis, and 10% body surface area) scores. Importantly, the study did not specify the ChatGPT model version or prompting strategy, substantially limiting interpretability and reproducibility.

Abate et al [20] evaluated the performance of ChatGPT and Gemini in applying the World Health Organization causality assessment algorithm to COVID-19 vaccine–associated myocarditis and pericarditis cases reported in the Vaccine Adverse Event Reporting System. Consistent with this study’s findings, ChatGPT demonstrated moderate agreement (71%) with human experts, whereas Gemini showed fair agreement (53%). In addition, both models failed to correctly recognize listed AEs in a nonnegligible proportion of cases, and Gemini exhibited pronounced inconsistency across repeated assessments. These results reinforce the observation that agreement with expert judgment and output stability are strongly influenced by model architecture, reasoning structure, and prompt design, and collectively underscore that current LLMs are best positioned as decision-support tools rather than replacements for expert-led causality assessment.

### Limitations

This study has several limitations that warrant consideration. First, the number of cases was limited (n=55), although a total of 337 drug-AE pair assessments were included. This relatively small case sample may not adequately capture the clinical complexity and heterogeneity of the broader FAERS database. In addition, multiple drug-AE assessments were derived from the same cases, potentially introducing intracase dependency and limiting the independence of observations. Consequently, the generalizability of the findings to real-world pharmacovigilance settings may be constrained. The dataset was constructed using a stratified sampling approach based on the number of suspected drugs per case, with equal representation across strata from 2 to 11 suspected drugs. This design was intended to evaluate model performance across a broad range of case complexities, including cases involving multiple suspected drugs. However, it does not reflect the natural distribution of FAERS reports, in which cases with fewer suspected drugs are generally more common. Therefore, the artificial enrichment of highly complex polypharmacy cases may have influenced the overall performance metrics and may limit their generalizability to routine pharmacovigilance workflows.

Second, class imbalance was present, with some categories (eg, “Conditional/Unclassified”) not represented and others underrepresented.

Third, causality assessment was conducted exclusively using the WHO-UMC framework. Although this framework is widely adopted in pharmacovigilance practice, it relies on categorical judgment rather than quantitative scoring. It was selected because it emphasizes qualitative clinical reasoning rather than predefined rules, making it more suitable for evaluating differences between LLM and expert assessments. Future studies should evaluate LLM performance across multiple causality assessment methods, including rule-based and probabilistic approaches, to enable a more comprehensive comparison.

Fourth, the cases were transformed into a semistructured format derived from FAERS structured fields, whereas real-world pharmacovigilance workflows often rely on unstructured narrative reports. Therefore, the model performance observed in this study may not fully generalize to the complexity and variability of raw individual case safety reports. However, this design allowed a controlled evaluation of causality reasoning by minimizing variability related to information extraction and data quality.

Fifth, the use of FAERS data introduces inherent structural limitations, including incomplete clinical information, reporting bias, and heterogeneity in report quality. In addition, a potential risk of data leakage due to possible overlap between publicly available FAERS reports and the training data of the evaluated LLMs cannot be fully excluded. However, FAERS does not provide explicit case-level causality assessments; therefore, the task required integration of multiple structured elements, including drug exposure, temporal relationships, and dechallenge or rechallenge information, rather than direct retrieval of preexisting causality labels. Accordingly, even if partial data overlap existed, model performance would be unlikely to reflect simple memorization alone and should instead be interpreted as involving multistep clinical reasoning across disparate case fields.

Finally, although expert consensus was used as the reference standard, interexpert disagreement remains an inherent challenge in causality assessment. Although a pilot consensus process was conducted to harmonize expert judgments, residual subjectivity cannot be fully eliminated.

Future research should explore hybrid approaches that combine LLMs with rule-based or algorithmic causality assessment methods. Such integrative frameworks may leverage the strengths of LLMs in handling unstructured clinical narratives while maintaining the transparency and reproducibility of established rule-based systems. In addition, expanding evaluation across multiple causality frameworks, languages, and real-world regulatory workflows will be essential to determine the practical role of LLMs in pharmacovigilance and regulatory decision-making.

### Conclusions

LLMs demonstrated moderate to substantial agreement in WHO-UMC drug-AE causality assessment, indicating meaningful but still limited performance relative to expert judgment. These findings suggest that LLMs are not yet ready for independent use in pharmacovigilance decision-making but may serve as supportive tools, particularly in preliminary case triage. Further studies using larger and more balanced datasets, as well as more rigorous evaluation of calibration, category-specific performance, and decision consistency, are needed to better define the role of LLMs in pharmacovigilance applications.

## Data Availability

The data used in this study were derived from the Food and Drug Administration Adverse Event Reporting System, which is publicly available. All curated cases used in this study, along with the full prompt templates applied for model evaluation, are provided in the multimedia appendices to ensure transparency and reproducibility.

## Appendix

Multimedia Appendix 1 Standardized input cases derived from the Food and Drug Administration Adverse Event Reporting System for large language model evaluation.

Multimedia Appendix 2 Full prompt templates for large language model–based causality assessment.

Multimedia Appendix 3 Performance comparison of large language models across prompting strategies for drug–adverse event causality assessment.

## Abbreviations

ABCD-10: age, bicarbonate, cancer, dialysis, and 10% body surface area
AE: adverse event
CoT: chain-of-thought
SC: self-consistency
FAERS: Food and Drug Administration Adverse Event Reporting System
LLM: large language model
WHO-UMC: World Health Organization–Uppsala Monitoring Centre
