# Supplementary material for: Large Language Models for World Health Organization–Uppsala Monitoring Centre Drug–Adverse Event Causality Assessment Using Food and Drug Administration Adverse Event Reporting System Cases: Comparative Performance Study
Source: J Med Internet Res. 2026 Jul 8;28:e93237. doi: 10.2196/93237 (PMC13392529; doi:10.2196/93237)
Supplement: Multimedia Appendix 2 [file jmir_v28i1e93237_app2.docx]

**Multimedia Appendix 2.** Full prompt templates for LLM-based causality assessment

**Section 1. Prompt structure**

| [General instruction]  You are a pharmacovigilance expert responsible for assessing drug–adverse event causality.  Please evaluate each drug (by its listed Drug Number) independently for its causality with the reported adverse events, according to WHO-UMC criteria:  (Certain, Probable/Likely, Possible, Unlikely, Conditional/Unclassified, Unassessable/Unclassifiable).  Even if drugs share the same active ingredients, their causality assessments must be evaluated independently.  [Critical interpretation guidance]  - “Certain” should be assigned when there is a definitive, well-recognized pharmacological event AND positive rechallenge.  - Distinguish “Probable” vs “Possible” carefully:    “Probable” does NOT require dechallenge data if temporal relationship is reasonable and alternative causes are unlikely.  - Assign “Probable” whenever the drug is the most likely candidate compared to other alternatives.  - Do NOT downgrade to “Possible” solely due to missing dechallenge information.  - “Possible” requires a plausible causal link, not just temporal alignment.  - “Possible” should ONLY be used when alternative explanations are equally or more plausible than the drug.  - Assign “Unlikely” if a stronger alternative explanation exists, even when temporal relationship is present.  - Use a stepwise approach: Start from “Possible” or “Probable”, then move upward if evidence is strong, or downward if evidence is weak. Select the category that best fits the overall evidence.  *{Strategy-specific instructions inserted here; see Section 2. (Strategy-specific prompt variants) below}*  [Case information]  *{Case-specific information inserted here; see Multimedia Appendix 1 for full reformatted cases}*  [Output format]  \|Drug Number\|Drug\|WHO-UMC Causality Assessment\|Reasoning Summary\|  Return ONLY the final table above.  Do NOT include any explanations, reasoning, analysis, or text before or after the table. |
| --- |

**Section 2. Strategy-specific prompt variants**

The following sections describe the strategy-specific instructions inserted into the prompt template depending on the prompting approach.

**2.1. Base:** No additional instructions

**2.2. CoT**

| [CoT]  Please reason through the following steps:  1. Temporal relationship: Is timing plausible?  2. Pharmacological plausibility: Is the event known/expected?  3. Alternative explanations: Disease? Other drugs?  4. Dechallenge / Rechallenge: Improvement after withdrawal? Reappearance?  5. Classification: Map findings to WHO-UMC category |
| --- |

**2.3. CoT-SC**

| Please reason through the following steps:  1. Generate at least three independent reasoning chains.  2. Each chain evaluates Temporal relationship, Pharmacological plausibility, Alternative explanations, and Dechallenge / Rechallenge.  3. Select the most consistent classification (majority or strongest evidence) |
| --- |

**2.4. Few-shot**

| [Few-shot]  Example:  Patient Information: Age 72 YR,Gender M.  Medications:  Drug1: GENTAMICIN, Dose: 160MG, Route: Intravenous, Indication: Post-op infection, Start Date: 20260401, End Date: 20260405 (abated dechallenge, recurred rechallenge)  Drug2: KETOROLAC, Dose: 30MG, Route: Intravenous, Indication: Pain control, Start Date: 20260401, End Date: 20260405 (abated dechallenge)  Drug3: RAMIPRIL, Dose: 5MG, Route: Oral, Indication: Hypertension, Start Date: 20250101, End Date: 20260405 (abated dechallenge)  Drug4: FAMOTIDINE, Dose: 20MG, Route: Oral, Indication: Stress ulcer prophylaxis, Start Date: 20260401, End Date: 20260405  Drug5: New Investigational Drug, Dose: 10MG, Route: Oral, Indication: Clinical trial, Start Date: 20260325, End Date: 20260405 (abated dechallenge)  Drug6: Herbal Supplement, Dose: N/A, Route: Oral, Indication: General health, Start Date: 20260325, End Date: 20260405  Adverse Reaction: Renal impairment.  Timeline of Events: Adverse reaction occurred on 20260405.  Assessment:  \|Drug Number\|Drug\|WHO-UMC Causality Assessment\|Reasoning Summary\|  \|1\| GENTAMICIN \| Certain \| The temporal relationship between drug administration and the onset of the adverse event is highly plausible. The recovery upon withdrawal (Positive Dechallenge) and the recurrence of the event upon re-exposure (Positive Rechallenge) provide definitive evidence. The reaction cannot be explained by the patient's underlying disease or other medications. \|  \|2\| KETOROLAC \| Probable/Likely \| There is a reasonable time sequence from the administration of the drug to the onset of the reaction. The event follows a known response pattern to this drug class (NSAID-induced nephrotoxicity) and improved significantly when the drug was stopped. While a rechallenge was not performed, the influence of underlying diseases or other drugs is unlikely to be the primary cause. \|  \|3\| RAMIPRIL \| Possible \| The time sequence is consistent with the event. However, the patient had been on this medication long-term. The AKI could easily be explained by other factors, such as the introduction of new nephrotoxic agents (Drug 1 and 2) or post-operative dehydration. While the condition improved after stopping the drug, the causal role of this specific medication remains uncertain due to these competing factors. \|  \|4\| FAMOTIDINE \| Unlikely \| While there is a temporal association, the pharmacological mechanism of this drug is not known to be associated with acute renal impairment. Other concurrent medications provide a much more compelling explanation for the reaction. \|  \|5\| New Investigational Drug \| Conditional/Unclassified \| This is a newly developed drug with a limited safety profile. While the timing of the AKI is suspicious, more data (such as additional case reports or literature regarding its mechanism) are required to make a definitive assessment. The causality is deferred until more evidence is available. \|  \|6\| Herbal Supplement \| Unassessable/Unclassifiable \| The patient reported taking an unidentified herbal supplement provided by a family member. Essential information—including the exact ingredients—is missing. Consequently, the report contains insufficient information to allow for a valid causality assessment. \| |
| --- |

**2.5. ReAct**

| Repeat until stable conclusion:  - Thought: Analyze Temporal relationship, Pharmacological plausibility, Alternative explanations, and Dechallenge / Rechallenge  - Action: Check alignment with WHO-UMC categories  - Observation: Identify inconsistencies or missing data  - Refinement: Adjust classification accordingly |
| --- |

**2.6. ToT**

| Please reason through the following steps:  1. Generate multiple reasoning paths.  2. Compare paths.  3. Select the most robust conclusion. |
| --- |
