# Supplementary material for: Large Language Models for World Health Organization–Uppsala Monitoring Centre Drug–Adverse Event Causality Assessment Using Food and Drug Administration Adverse Event Reporting System Cases: Comparative Performance Study
Source: J Med Internet Res. 2026 Jul 8;28:e93237. doi: 10.2196/93237 (PMC13392529; doi:10.2196/93237)
Supplement: Multimedia Appendix 3 [file jmir_v28i1e93237_app3.docx]

**Multimedia Appendix 3.** Performance comparison of LLMs across prompting strategies for drug–AE causality assessment

| Model | Prompt | Cohen’s κ | Weighted κ | Accuracy | Balanced accuracy | Fleiss’ κ |
| --- | --- | --- | --- | --- | --- | --- |
| Gemini 2.5 Pro | Base | 0.483 (0.418, 0.543) | 0.689 (0.616, 0.751) | 0.676 (0.630, 0.721) | 0.713 (0.687, 0.740) | 0.794 |
|  | CoT | 0.487 (0.423, 0.544) | 0.687 (0.615, 0.749) | 0.684 (0.639, 0.723) | 0.725 (0.703, 0.747) | 0.739 |
|  | CoT-SC | **0.514 (0.451, 0.574)** | **0.700 (0.630, 0.756)** | **0.704 (0.661, 0.745)** | **0.735 (0.715, 0.758)** | 0.758 |
|  | Few-shot | 0.471 (0.407, 0.532) | 0.681 (0.612, 0.741) | 0.668 (0.620, 0.712) | 0.711 (0.690, 0.732) | **0.795** |
|  | ReAct | 0.444 (0.377, 0.502) | 0.663 (0.588, 0.727) | 0.648 (0.602, 0.691) | 0.699 (0.678, 0.721) | 0.771 |
|  | ToT | 0.482 (0.421, 0.545) | 0.652 (0.570, 0.717) | 0.677 (0.634, 0.720) | 0.722 (0.699, 0.744) | 0.733 |
| Gemini 2.5 Flash | Base | 0.611 (0.547, 0.673) | 0.813 (0.763, 0.859) | 0.783 (0.745, 0.820) | 0.674 (0.632, 0.716) | 0.862 |
|  | CoT | **0.641 (0.579, 0.701)** | 0.819 (0.766, 0.864) | 0.803 (0.766, 0.840) | 0.707 (0.664, 0.748) | 0.846 |
|  | CoT-SC | 0.640 (0.572, 0.708) | **0.821 (0.767, 0.865)** | **0.804 (0.763, 0.845)** | **0.723 (0.690, 0.753)** | **0.915** |
|  | Few-shot | 0.562 (0.491, 0.628) | 0.759 (0.691, 0.816) | 0.747 (0.705, 0.789) | 0.714 (0.695, 0.732) | 0.851 |
|  | ReAct | 0.619 (0.556, 0.679) | 0.820 (0.771, 0.862) | 0.792 (0.755, 0.827) | 0.676 (0.633, 0.715) | 0.854 |
|  | ToT | 0.625 (0.561, 0.686) | 0.791 (0.732, 0.841) | 0.792 (0.753, 0.828) | 0.702 (0.654, 0.745) | 0.849 |
| GPT 5.4 | Base | 0.467 (0.406, 0.531) | 0.680 (0.609, 0.742) | 0.674 (0.628, 0.719) | 0.615 (0.558, 0.666) | 0.838 |
|  | CoT | 0.479 (0.415, 0.541) | 0.696 (0.630, 0.752) | 0.680 (0.631, 0.723) | 0.590 (0.539, 0.641) | 0.856 |
|  | CoT-SC | 0.501 (0.434, 0.565) | 0.709 (0.641, 0.768) | 0.696 (0.649, 0.740) | 0.570 (0.519, 0.621) | 0.883 |
|  | Few-shot | 0.495 (0.428, 0.556) | **0.728 (0.663, 0.780)** | 0.686 (0.640, 0.731) | **0.716 (0.688, 0.742)** | **0.884** |
|  | ReAct | **0.507 (0.445, 0.569)** | 0.712 (0.646, 0.770) | **0.697 (0.652, 0.741)** | 0.622 (0.566, 0.672) | 0.846 |
|  | ToT | 0.482 (0.419, 0.544) | 0.700 (0.634, 0.757) | 0.682 (0.636, 0.728) | 0.582 (0.531, 0.632) | 0.869 |
| GPT 5.4 mini | Base | 0.388 (0.323, 0.451) | 0.648 (0.571, 0.715) | 0.617 (0.570, 0.662) | 0.513 (0.468, 0.564) | 0.791 |
|  | CoT | **0.435 (0.366, 0.500)** | 0.651 (0.571, 0.720) | **0.661 (0.617, 0.706)** | 0.538 (0.487, 0.586) | 0.820 |
|  | CoT-SC | 0.434 (0.368, 0.495) | 0.663 (0.585, 0.729) | 0.642 (0.599, 0.685) | 0.582 (0.530, 0.633) | 0.730 |
|  | Few-shot | 0.368 (0.305, 0.431) | 0.641 (0.566, 0.708) | 0.583 (0.535, 0.631) | **0.587 (0.533, 0.637)** | 0.775 |
|  | ReAct | 0.415 (0.346, 0.479) | 0.643 (0.559, 0.715) | 0.641 (0.594, 0.685) | 0.517 (0.467, 0.566) | 0.805 |
|  | ToT | 0.434 (0.369, 0.495) | **0.668 (0.594, 0.736)** | 0.648 (0.602, 0.691) | 0.547 (0.501, 0.594) | **0.834** |

CoT, chain-of-thought; CoT-SC, chain-of-thought with self-consistency; ReAct, reasoning and acting; ToT, tree-of-thoughts

For each metric, bold values indicate the highest score within each model, while underlined values indicate the highest score across all models.
